# Photoclick Reaction Constructs Glutathione-Responsive Theranostic System for Anti-Tuberculosis

**DOI:** 10.3389/fmolb.2022.845179

**Published:** 2022-02-14

**Authors:** Judun Zheng, Xun Long, Hao Chen, Zhisheng Ji, Bowen Shu, Rui Yue, Yechun Liao, Shengchao Ma, Kun Qiao, Ying Liu, Yuhui Liao

**Affiliations:** ^1^ Molecular Diagnosis and Treatment Center for Infectious Diseases, Dermatology Hospital, Southern Medical University, Guangzhou, China; ^2^ Department of Science and Education, The Third People’s Hospital of Bijie City, Bijie, China; ^3^ Division of Gastrointestinal Surgery, Department of General Surgery, Nanfang Hospital, Southern Medical University, Guangzhou, China; ^4^ Department of Orthopedics, The First Affiliated Hospital of Jinan University, Jinan University, Guangzhou, China; ^5^ NHC Key Laboratory of Metabolic Cardiovascular Diseases Research, Ningxia Key Laboratory of Vascular Injury and Repair Research, Ningxia Medical University, Yinchuan, China; ^6^ Department of Thoracic Surgery, Shenzhen Third People’s Hospital, Shenzhen, China; ^7^ Department of Infectious Disease, The Fifth Affiliated Hospital, Sun Yat-sen University, Guangzhou, China

**Keywords:** tuberculosis, theranostic system, photoclick reaction, microenvironment, glutathione

## Abstract

Tuberculosis (TB) is a virulent form of an infectious disease that causes a global burden due to its high infectivity and fatality rate, especially the irrepressible threats of latent infection. Constructing an efficient strategy for the prevention and control of TB is of great significance. Fortunately, we found that granulomas are endowed with higher reducibility levels possibly caused by internal inflammation and a relatively enclosed microenvironment. Therefore, we developed the first targeted glutathione- (GSH-) responsive theranostic system (RIF@Cy5.5-HA-NG) for tuberculosis with a rifampicin- (RIF-) loaded near-infrared emission carrier, which was constructed by photoclick reaction-actuated hydrophobic-hydrophobic interaction, enabling the early diagnosis of tuberculosis through granulomas-tracking. Furthermore, the loaded rifampicin was released through the dissociation of disulfide bond by the localized GSH in granulomas, realizing the targeted tuberculosis therapy and providing an especially accurate treatment mapping for tuberculosis. Thus, this targeted theranostic strategy for tuberculosis exhibits the potential to realize both granulomas-tracking and anti-infection of tuberculosis.

## Introduction

As tuberculosis (TB) is highly contagious, it places a heavy burden on public health worldwide (Pai et al., 2016; Dai et al., 2020; Zwerling, 2020; Daftary et al., 2021; Xu et al., 2021). This chronic disease caused by *Mycobacterium tuberculosis* (M. tb) most often affects the lungs (Arcos et al., 2017; Rothchild et al., 2019; Fernández-García et al., 2020; Allue-Guardia et al., 2021). Tuberculosis also arises in other organs, including bone and spine, then evolving into one of the most typical forms of extrapulmonary tuberculosis (Pandey et al., 2009; Magnussen et al., 2013; Pigrau-Serrallach and Rodríguez-Pardo, 2013; Rodriguez-Takeuchi et al., 2019). Recently, the surgical intervention accompanied by indispensable anti-tubercular drug therapy is the primary routine treatment (Mukewar et al., 2012; Shrivastava et al., 2019; Moosa et al., 2020; Phillips et al., 2020). Although rifampicin (RIF) and isoniazid have been widely chosen as clinical anti-tubercular drugs due to their excellent effectiveness and reasonable price (Hakkimane et al., 2018; Campbell et al., 2020; Sterling et al., 2020; Villa et al., 2020; Kabir et al., 2021), their short plasma life and relatively low concentration in tuberculosis granulomas and the inescapable side effects of chemotherapeutic drugs have drawn growing attention from interdisciplinary and clinical medicine research circles (Du Toit et al., 2006; Dartois, 2014; Liao et al., 2020). Thus, there is an urgent need to develop an efficient chemotherapy strategy for tuberculosis.

As the typical lesion core of tuberculosis, granuloma formation provides a relatively closed space that could prevent the entrance of anti-tuberculosis drugs (Ramakrishnan, 2012; Ehlers and Schaible, 2013; Bhavanam et al., 2016). Few drugs can penetrate the central regions due to the compact structure of granulomas, and the non-growing bacteria inside the granulomas are inherently recalcitrant to killing by most antibiotics (Datta et al., 2015; Sarathy et al., 2016). The infection of M. tb can remain “silent” throughout an individual’s life but can be reactivated by various conditions to stimulate new bacterial growth and infect new patients, even after decades (Ahmad, 2010; Gengenbacher and Kaufmann, 2012). This is why patients with TB require lengthy multidrug therapy, which would increase the risk of multidrug resistance. On the one hand, nanotechnology, possessing intriguing physicochemical and biophysical properties, such as high surface area-to-volume ratio, multifunctionality and controllable release, simple synthesis methods, and lower eco-toxicity, offers a promising alternative in the theranostics of tumor and neuroscience (Kumar et al., 2017; Feng et al., 2020; Fu et al., 2020). On the other hand, as we reported in ACS Nano (Liao et al., 2020), the granuloma formation possesses the effect of enhanced permeability and retention, which provides the possibility for targeted diagnosis and therapy.

**SCHEME 1:**
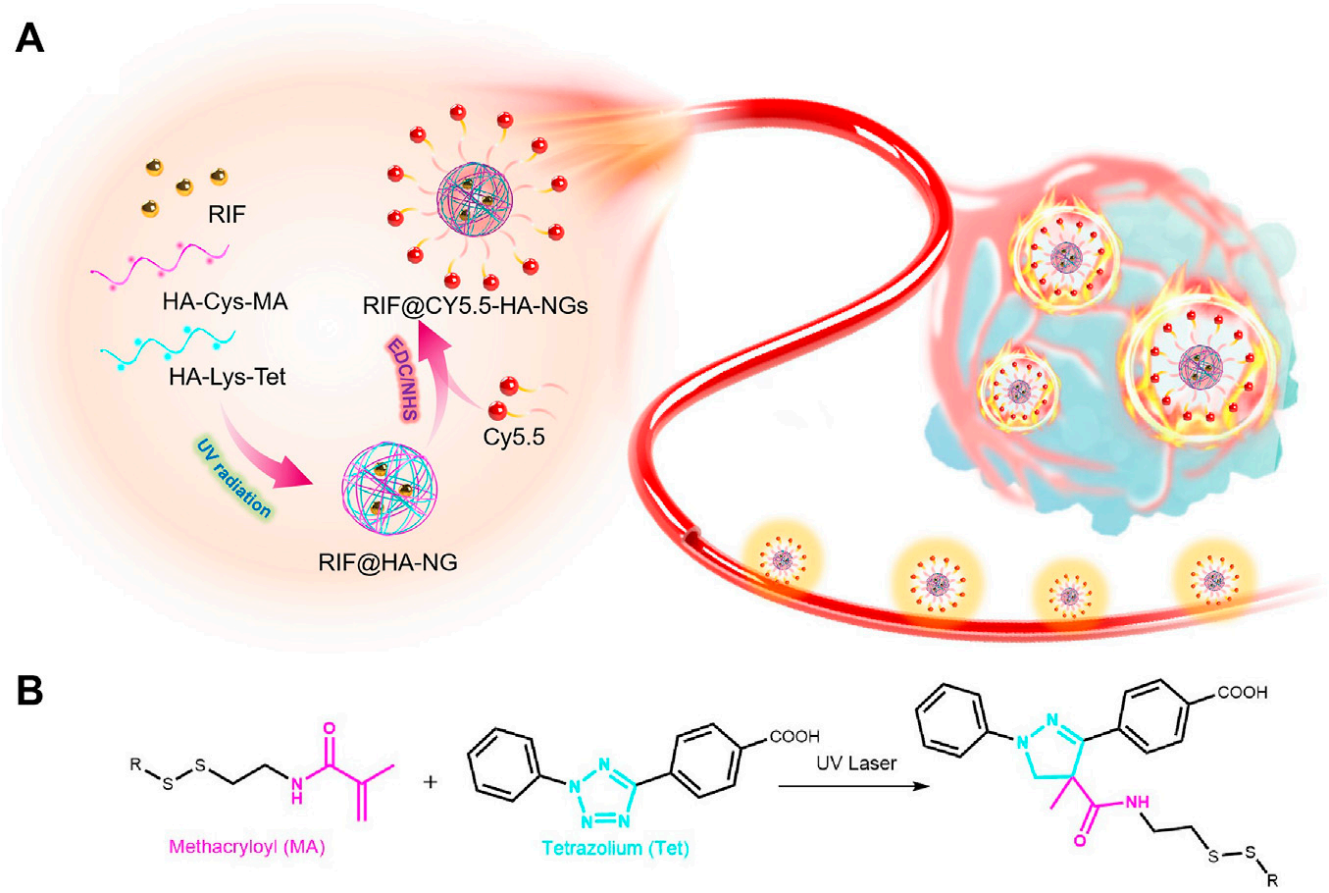
Scheme of photoclick reaction constructing glutathione-responsive theranostic system for anti-tuberculosis. **(A)** Schematic illustration of the constructing glutathione-responsive theranostic nanoagents for anti-tuberculosis. **(B)** The photoclick chemical reaction between methacryloyl (MA) and tetrazolium (Tet).

Fortunately, we have found that granulomas are endowed with relatively reducibility levels possibly caused by internal inflammation and relatively enclosed microenvironments (Kiran et al., 2016; Muefong and Sutherland, 2020; Singh et al., 2020). Click chemistry is a powerful linking reaction that is simple to manipulate, possesses high yields, and is versatile in joining diverse structures without the prerequisite of protection steps (Hein et al., 2008; Nwe and Brechbiel, 2009; Zhang et al., 2020; Zheng et al., 2020). In particular, photo-induced tetrazole-based click chemistry has been exploited widely as an efficient tool for site-selective modification or optimization of proteins (Herner and Lin, 2016; Shang et al., 2017; Wu et al., 2019). Therefore, we developed the first targeted glutathione- (GSH-) responsive theranostic system (RIF@Cy5.5-HA-NG) for tuberculosis with a rifampicin- (RIF-) loaded near-infrared emission carrier, which was constructed by photoclick reaction-actuated hydrophobic-hydrophobic interaction, enabling the early diagnosis of tuberculosis through granulomas-tracking (Scheme 1). The constructed GSH-activatable RIF@Cy5.5-HA-NG realized the M. tb-selective imaging, affording precise and effective inhibition of the localized tuberculosis *via* released RIF for the synergistic treatment of persistent bacteria. This work demonstrated that the rifampicin-loaded GSH-activatable hyaluronic acid (HA) system is a reliable tool for effective tuberculosis therapy.

## Experimental Section

### Materials

1-(3-Dimethylaminopropyl)-3-ethylcarbodiimide hydro (EDC), N-hydroxy succinimide (NHS), cysteine (Cys), hyaluronic acid (HA), and methacryloyl chloride (MA) were purchased from Aladdin Reagent, Ltd. (Shanghai, China). All chemicals used in this study were of analytical grade, and reagents were used without further purification. Dulbecco’s modified Eagles medium (DMEM) cell culture medium, fetal bovine serum, streptomycin, and penicillin were purchased from Thermo Fisher Scientific Co., Ltd. (China). All aqueous solutions were prepared using ultrapure deionized water (DI water), which was obtained through a Millipore Milli-Q water purification system (Billerica, United States) and had an electric resistance >18.2 MΩ.

### Methods

The UV–vis absorption spectra were recorded on a UV–vis spectrometer (Lambda 35 UV–vis spectrometer, Perkin-Elmer, United States) at room temperature. The sizes of micelle nanoparticles were measured using ZEN3690 zetasizer (Malvern instruments, Zetasizer Nano-ZS) at room temperature. The confocal fluorescence images of the cell were collected on a Zeiss Laser Scanning Confocal Microscope (ZEISS LSM 780, Germany). *In vivo* fluorescence images were captured by the Xenogen IVIS Spectrum system (Caliper Life Sciences, Hopkinton, MA).

### The Construction of Granuloma-Bearing Mouse Models

All animal experiments were approved by the Institutional Animal Ethics Committee at Southern Medical University, and the experiments were performed in compliance with the National Institutes of Health (NIH) guidelines for the Care and Use of Laboratory Animals of Southern Medical University. The granuloma-bearing mouse models were in accordance with the previous model reported according to the literature (Carlsson et al., 2010; Oehlers et al., 2015; Fenaroli et al., 2018; Liao et al., 2020). Briefly, female C57BL/6 (B6) mice (8–12 weeks old) were purchased from Guangdong Medical Laboratory Animal Center (Guangdong, China). To establish the granuloma-bearing mouse models, *Mycobacterium marinum* (M.m) were first grown in 7H9 broth media for 7–10 days in a bacterial shaking incubator. When the mid-log phase (OD600 = 0.7 ± 0.2) was achieved, the bacteria were collected, washed, and then passed 20 times through a needle to disrupt bacterial aggregates. After that, the supernatants were transferred to new tubes and diluted in sterile PBS to a final concentration of 2 × 108 CFU ml^-1^. Two hundred microliters of the M.m suspension was intravenously injected into mice using an insulin syringe. The bacteria infection-induced tail granulomas were successfully formed after 2 weeks.

## Results and Discussion

### Preparation of RIF@HA-NG

In order to realize the theranostic effect on tuberculosis, the GSH-responsive nanoagent was rationally designed for targeted imaging and therapy of tuberculosis. RIF@Cy5.5-HA-NG was first synthesized between two types of extensively biocompatible hyaluronic acid (HA) as the host material and near-infrared dye Cy5.5 as a contrast agent via the photo-initiated bioorthogonal reaction and the hydrophilic-hydrophilic interaction. Furthermore, the loaded rifampicin was released through the dissociation of disulfide bonds by the original GSH in granulomas, realizing targeted tuberculosis therapy and providing especially accurate treatment mapping for tuberculosis. Glutathione, as a tripeptide and antioxidant, is synthesized at high levels under intracellular oxidative stress (Aquilano et al., 2014; Zheng et al., 2019a), playing an important role in apoptosis and regulating pathogen-infected-host interaction, including inhibition of *M. tuberculosis* replication (Seres et al., 2000; Venketaraman et al., 2003; Venketaraman et al., 2005). The resulting system was characterized for GSH-responsive rifampicin release, real-time monitoring, and antibiosis properties. Then, the prolonged retention time of drug-release *in vitro* and the *in vivo* was demonstrated using fluorescence imaging techniques.

In this study, HA-Cys-MA and HA-Lys-Tet, which could first form nanocages *via* UV-induced click reaction, were mixed with rifampicin (RIF) to create a RIF-loaded carrier (RIF@HA-NG). Photo-inducible click chemistry has been widely applied to functionalize and investigate the dynamics and roles of biomolecules in living systems (Le Droumaguet et al., 2010; Zhou et al., 2016; Nainar et al., 2017; Liu et al., 2021). The fluorescent imaging contrast Cy5.5 was then modified on the RIF@HA-NG through amidation in the existence of carbodiimide (EDC) and N-hydroxysuccinimide (NHS) to obtain the aimed nanosystem, which combined the diagnosis and therapy of tuberculosis (Scheme 1). Among them, the synthetic routes of HA-Cys-MA and HA-Lys-Tet are summarized in Supplementary Figures S1, S2, respectively. On the one hand, GSH plays an important role in many diseases, including cancer and tuberculosis (Allen et al., 2015; Zheng et al., 2019b). Moreover, the cysteine (Cys) containing disulfide bond (Yang et al., 2015; Wang et al., 2020) was reasonably chosen to possess the GSH-responsive peculiarity. On the other hand, a polymer pre-monomer containing the photo-click functional groups, including methacryloyl (MA) (Maiti et al., 2020) and tetrazolium (Tet) (Wu et al., 2019; Liu et al., 2021), was designed and synthesized to obtain a controllable nano-delivery system.

### Characteristics of RIF@HA-NG

**FIGURE 1:**
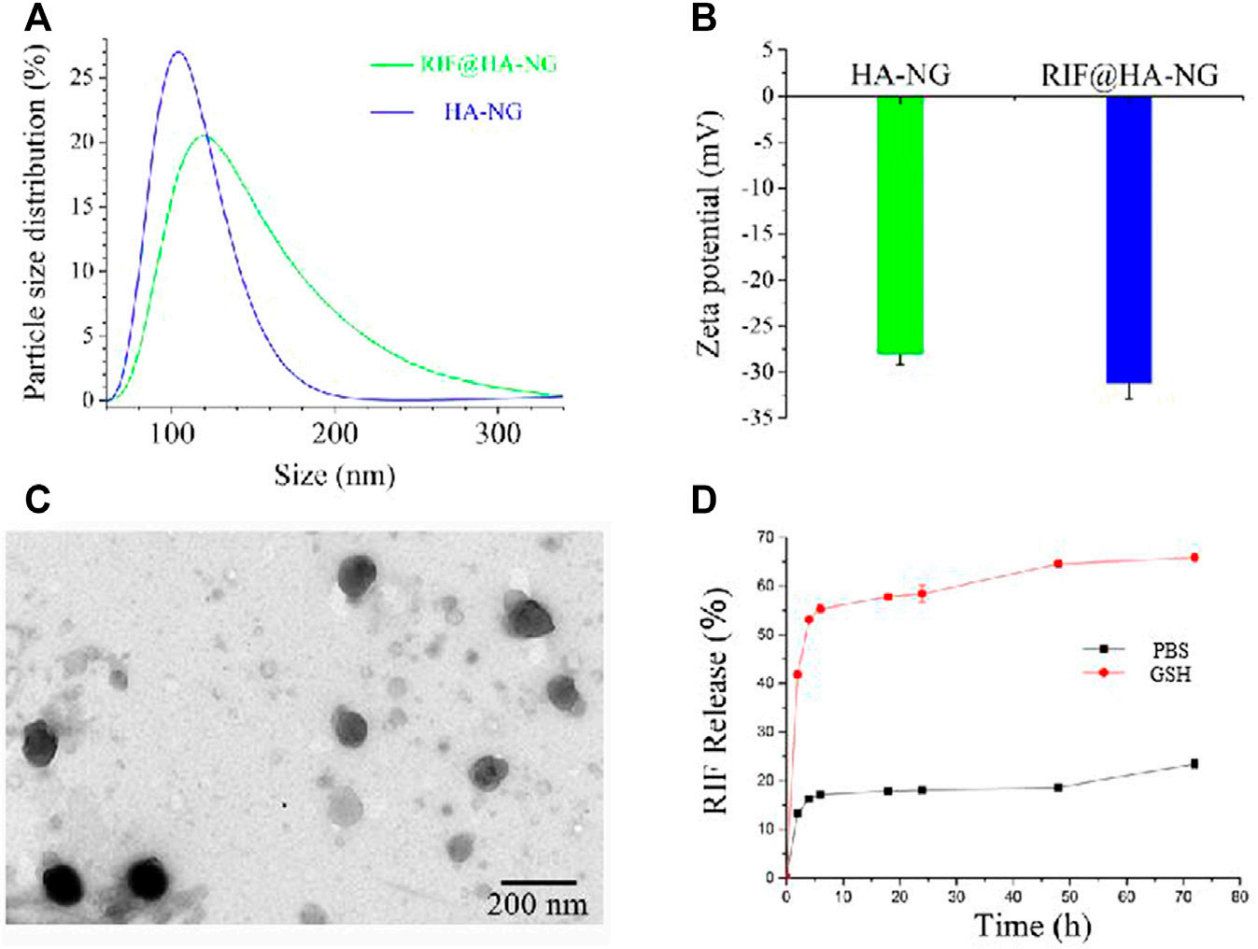
Characterizations of the RIF@HA-NG. **(A)** The dynamic light scattering (DLS) analysis of HA-NG and RIF@HA-NG. **(B)** The zeta potential results of HA-NG and RIF@HA-NG. **(C)** The transmission electron microscopy (TEM) images of RIF@HA-NG, scale bars are 200 nm. **(D)** The RIF release of RIF@HA-NG (PBS) in the absence or presence of GSH.

The synthesis of RIF@HA-NG was first characterized by the dynamic light scattering (DLS) analyzer. As shown in Figure 1A, the RIF@HA-NG nanoagent had a hydrodynamic diameter of approximately 120 nm, which was slightly larger than that of HA-NG owning to the RIF loading (Figure 1A). To further confirm the successful loading of RIF, the zeta potentials were measured, showing the zeta potential change from −27.5 to −31.3 mV. In other words, the negative RIF obviously decreased the zeta potential of the nanoagent RIF@HA-NG (Figure 1B). Transmission electron microscopy (TEM) revealed that RIF@HA-NG exhibited a uniform morphology and size with a diameter of 100 nm, indicating that no obvious changes were recorded in the size and shape of the nanoagent after loading RIF (Figure 1C). Meanwhile, the drug-release ability of nanocarriers was assessed, so the RIF release analysis was explored (Figure 1D). Obviously, the ∼55% of the total release was observed within 10 h, and the total release reached ∼70% upon GSH treatment for 70 h. In contrast, the total release reached only ∼20% in PBS solution up to 70 h. *In vitro* GSH-triggered drug-release analysis indicated that a greater amount of RIF can be released in *Mycobacterium*-infected macrophage cells. Collectively, these results confirmed that the GSH-responsive water-soluble RIF@HA-NG was successfully synthesized.

### Antibacterial Activity of RIF and RIF@Cy5.5-HA-NG

**FIGURE 2:**
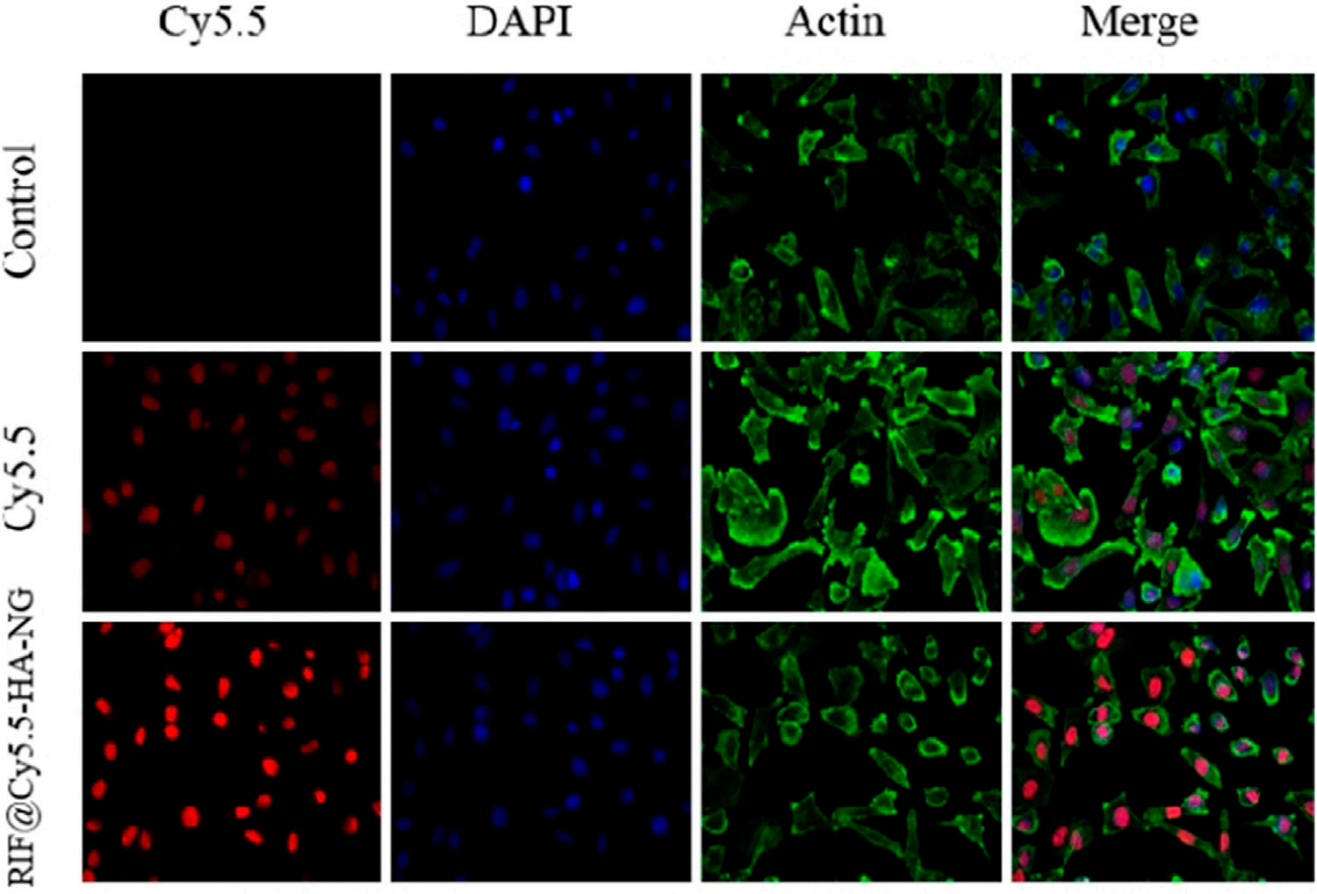
Uptake of RIF@Cy5.5-HA-NG. Blue fluorescent image indicates the image of DAPI stained cells. Red fluorescence indicates uptake of Cy5.5 and RIF@Cy5.5-HA-NG. Green fluorescent image indicates the image of Actin. To demonstrate the uptake of RIF@Cy5.5-HA-NG nanoparticles, the laser confocal experiment was performed and the section-wise imaging of intracellular localization of nanoparticles was shown.

With the nanoagent in hand, we then aimed to estimate the deliverability of RIF in targeting cells. To determine whether the RIF-loaded nanoagent was endocytosed by the macrophage cell, the RIF@HA-NG was modified by the near-infrared fluorescence dye Cy5.5, simultaneously realizing the imaging of tuberculosis. As shown in Figure 2, the red fluorescence was detected in the RIF@Cy5.5-HA-NG-treated group compared to the Cy5.5-treated group, indicating that RIF@HA-NG can accumulate in the granuloma. That is to say, RIF@HA-NG was successfully modified by Cy5.5. Thus, it can be rationally used to monitor tuberculosis.

**FIGURE 3:**
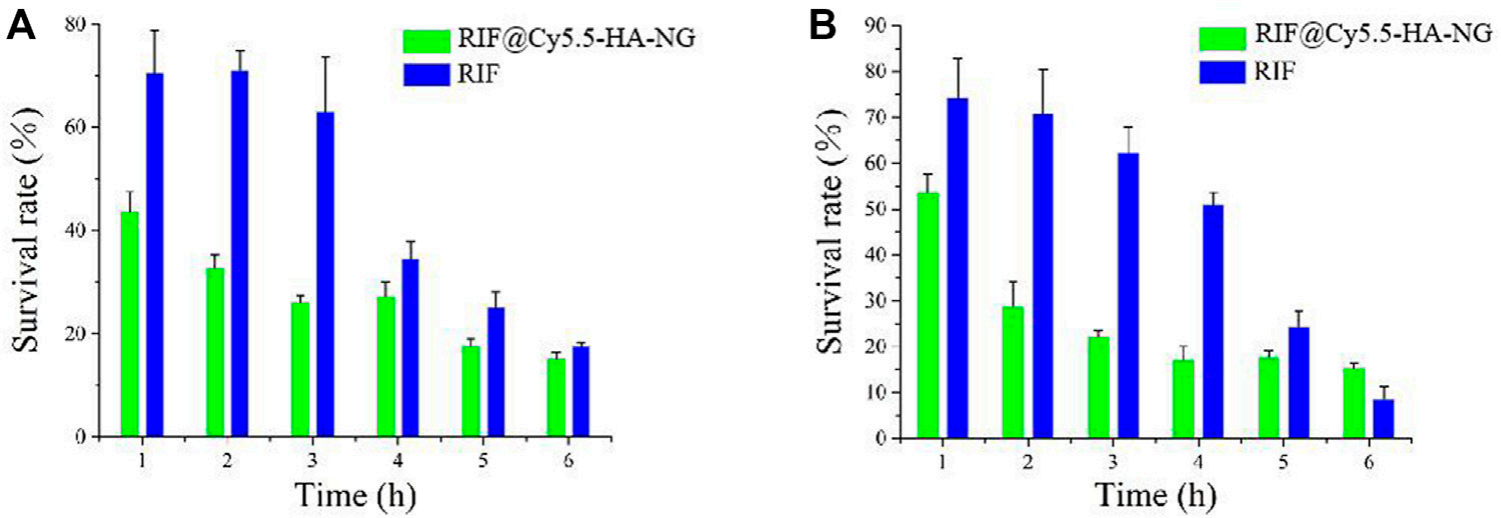
Antibacterial activity of RIF and RIF@Cy5.5-HA-NG *in vitro*. Survival analysis of **(A)** macrophage cells (M1) and **(B)** macrophage cells (M2) with RIF and RIF@Cy5.5-HA-NG.

Owing to the outstanding drug-release properties of RIF@Cy5.5-HA-NG toward GSH-enriched tuberculosis, we then investigated its antibacterial performance. To investigate the antibacterial activity of RIF and RIF@HA-NG *in vitro*, M. tb-infected M1 macrophage cells were first incubated with RIF and RIF@Cy5.5-HA-NG at different times. Survival analysis was performed to confirm the antibacterial effects of RIF and RIF@Cy5.5-HA-NG. As displayed in Figure 3A, the survival rate of the group treated with RIF@Cy5.5-HA-NG for 1–3 h was lower than that of the RIF-treated group. In particular, RIF@Cy5.5-HA-NG or RIF was co-incubated with cells for 3 h, and the survival rate of bacteria decreased to 28 and 63%, respectively. When the processing time was extended, the bacterial damage caused RIF@Cy5.5-HA-NG to increase, but the survival rate changed gently, which may have been caused by the phytocytosis of macrophages. Meanwhile, the survival analysis of RIF@Cy5.5-HA-NG and RIF treated with *Mycobacterium*-infected M2 macrophage cells also showed a similar antibacterial tendency, indicating the antibacterial activity of nanoparticles (NPs) was superior to that of pure RIF (Figure 3B). Taken together, these results performed that RIF@Cy5.5-HA-NG NPs have clipping high antibacterial efficiency against cellular bacteria *in vitro* and therefore hold potential for tuberculosis treatment.

### Evaluation of the Antibacterial Activity and Toxicity of the RIF@Cy5.5-HA-NG

**FIGURE 4:**
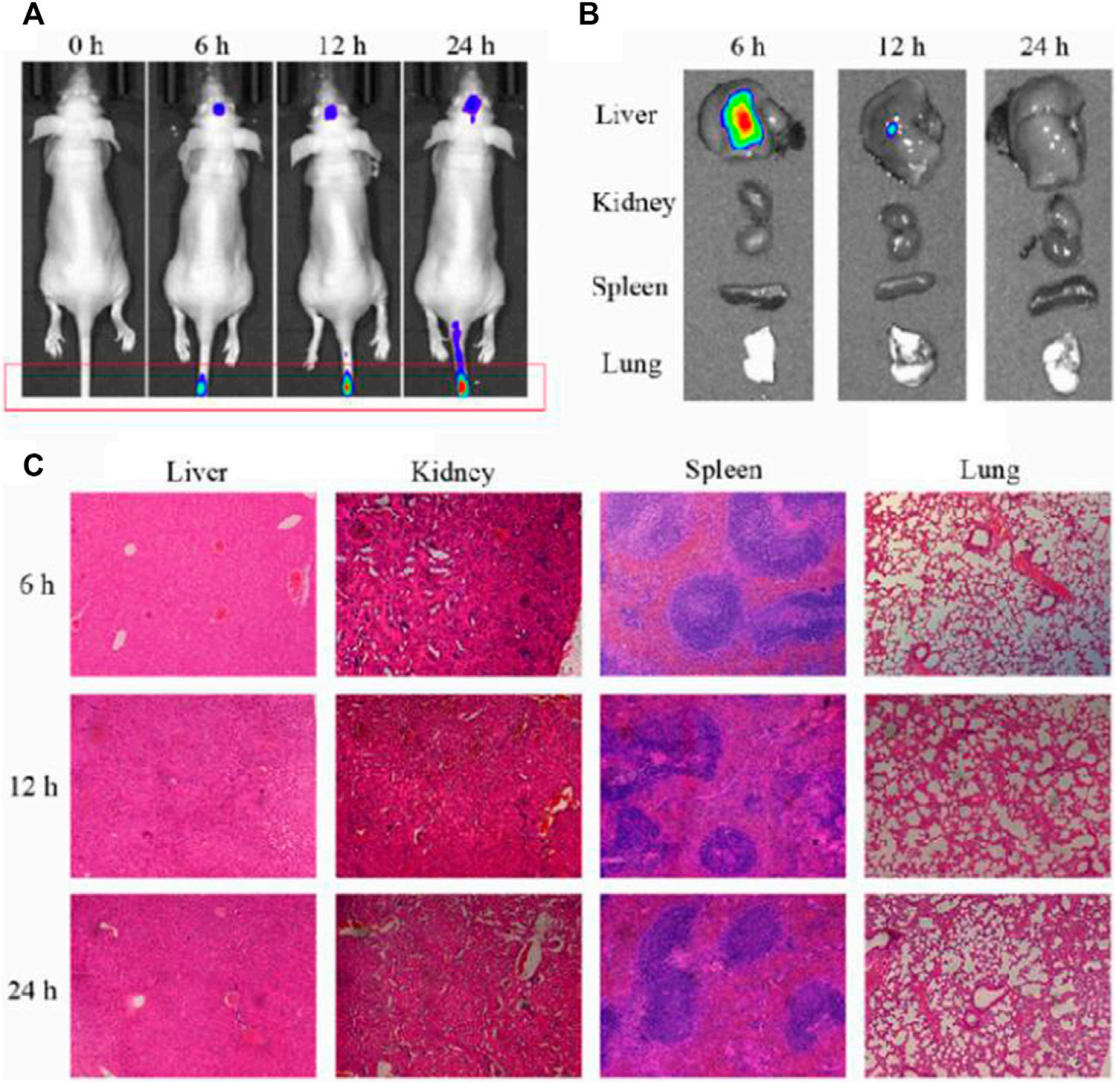
Evaluation of the antibacterial activity and toxicity of the RIF@Cy5.5-HA-NG. **(A)** Fluorescence imaging of mice at 0, 6, 12, and 24 h after injection. **(B)** Fluorescence imaging of major organs (liver, spleen, lung, and kidney) collected from animals at 0, 6, 12, and 24 h after injection. **(C)** Tissue damage analysis of different important organs in mice after intravenous injection of RIF@HA-NG at different times.

Having established that RIF@Cy5.5-HA-NG nanoagent could efficiently kill *Mycobacterium*, our next goal was to validate its potential to monitor the *Mycobacterium*-infected mice. First, we established the tuberculosis model by injecting *Mycobacterium marinum* into the tail vein of mice according to our previously reported methods (Liao et al., 2020). The mice were imaged under a 633 nm laser using the fluorescence *in vivo* imaging system at different times (0, 6, 12, and 24 h). As exhibited in Figure 4A, the fluorescence intensity at 690 nm in the granuloma region was found to reach the maximum at 24 h, indicating that the concentration of RIF@Cy5.5-HA-NG increased gradually over time and was maintained at a relatively high level even at 24 h after injection. To further investigate the distribution of nanoagent in various organs at 2, 4, and 24 h time points, *ex vivo* fluorescence imaging was also studied. Intense fluorescence signals at 690 nm were observed in the liver (Figure 4B), which indicated that the nanoagent was preferred to selectively accumulate in the liver. The selective accumulation may be due to the reticuloendothelial system (Zheng et al., 2019; Peng et al., 2017), which indicated that these nanoagents could be metabolized through the liver. Furthermore, hematoxylin and eosin (HE) staining of various important organs revealed no pathological changes after RIF@Cy5.5-HA-NG nanoagents injection at different time points (Figure 4C). Taken together, these results firmly demonstrate that RIF@Cy5.5-HA-NG is capable of directly reflecting tuberculosis, revealing the feasibility of our nanoagent for monitoring the mycobacterium *in vivo* as an excellent biomaterial.

## Conclusion

In summary, a hybrid material that combined the biocompatible hyaluronic acid and functional agents, including drug rifampicin and Dye Cy5.5, has been constructed and verified as effective mycobacterium tuberculosis-targeting nanoagent for targeted tuberculosis chemotherapy. The GSH-activatable RIF@Cy5.5-HA-NG exhibited not only excellent *Mycobacterium tuberculosis* targeting selectivity and biocompatibility but also a high anti-tuberculosis effect. Therefore, this study opened up new access to develop the tuberculosis-specific degradable nanomaterials for targeted imaging and therapy of tuberculosis.

## Data Availability

The original contributions presented in the study are included in the article/Supplementary Material. Further inquiries can be directed to the corresponding authors.

## References

[B1] Ahmad S. (2010). New Approaches in the Diagnosis and Treatment of Latent Tuberculosis Infection. Respir. Res. 11 (1), 169. 10.1186/1465-9921-11-169 21126375 PMC3004849

[B2] Allen M. Bailey C. Cahatol I. Dodge L. Yim J. Kassissa C. (2015). Mechanisms of Control of *Mycobacterium tuberculosis* by NK Cells: Role of Glutathione. Front. Immunol. 6, 508. 10.3389/fimmu.2015.00508 26500648 PMC4593255

[B3] Allué-Guardia A. García J. I. Torrelles J. B. (2021). Evolution of Drug-Resistant *Mycobacterium tuberculosis* Strains and Their Adaptation to the Human Lung Environment. Front. Microbiol. 12, 612675. 10.3389/fmicb.2021.612675 33613483 PMC7889510

[B4] Aquilano K. Baldelli S. Ciriolo M. R. (2014). Glutathione: New Roles in Redox Signaling for an Old Antioxidant. Front. Pharmacol. 5, 196. 10.3389/fphar.2014.00196 25206336 PMC4144092

[B5] Arcos J. Sasindran S. J. Moliva J. I. Scordo J. M. Sidiki S. Guo H. (2017). *Mycobacterium tuberculosis* Cell wall Released Fragments by the Action of the Human Lung Mucosa Modulate Macrophages to Control Infection in an IL-10-dependent Manner. Mucosal Immunol. 10 (5), 1248–1258. 10.1038/mi.2016.115 28000679 PMC5479761

[B6] Bhavanam S. Rayat G. R. Keelan M. Kunimoto D. Drews S. J. (2016). Understanding the Pathophysiology of the Human TB Lung Granuloma Using *In Vitro* Granuloma Models. Future Microbiol. 11 (8), 1073–1089. 10.2217/fmb-2016-0005 27501829

[B7] Campbell J. R. Trajman A. Cook V. J. Johnston J. C. Adjobimey M. Ruslami R. (2020). Adverse Events in Adults with Latent Tuberculosis Infection Receiving Daily Rifampicin or Isoniazid: post-hoc Safety Analysis of Two Randomised Controlled Trials. Lancet Infect. Dis. 20 (3), 318–329. 10.1016/S1473-3099(19)30575-4 31866327

[B8] Carlsson F. Kim J. Dumitru C. Barck K. H. Carano R. A. D. Sun M. (2010). Host-detrimental Role of Esx-1-Mediated Inflammasome Activation in Mycobacterial Infection. Plos Pathog. 6 (5), e1000895. 10.1371/journal.ppat.1000895 20463815 PMC2865529

[B9] Daftary A. Mondal S. Zelnick J. Friedland G. Seepamore B. Boodhram R. (2021). Dynamic Needs and Challenges of People with Drug-Resistant Tuberculosis and HIV in South Africa: a Qualitative Study. Lancet Glob. Health 9 (4), e479–e488. 10.1016/S2214-109X(20)30548-9 33740409 PMC8009302

[B10] Dai T. Xie J. Zhu Q. Kamariza M. Jiang K. Bertozzi C. R. (2020). A Fluorogenic Trehalose Probe for Tracking Phagocytosed *Mycobacterium tuberculosis* . J. Am. Chem. Soc. 142 (36), 15259–15264. 10.1021/jacs.0c07700 32813512 PMC9067319

[B11] Dartois V. (2014). The Path of Anti-tuberculosis Drugs: from Blood to Lesions to Mycobacterial Cells. Nat. Rev. Microbiol. 12 (3), 159–167. 10.1038/nrmicro3200 24487820 PMC4341982

[B12] Datta M. Via L. E. Kamoun W. S. Liu C. Chen W. Seano G. (2015). Anti-vascular Endothelial Growth Factor Treatment Normalizes Tuberculosis Granuloma Vasculature and Improves Small Molecule Delivery. Proc. Natl. Acad. Sci. USA 112 (6), 1827–1832. 10.1073/pnas.1424563112 25624495 PMC4330784

[B13] Du Toit L. C. Pillay V. Danckwerts M. P. (2006). Tuberculosis Chemotherapy: Current Drug Delivery Approaches. Respir. Res. 7 (1), 118. 10.1186/1465-9921-7-118 16984627 PMC1592088

[B14] Ehlers S. Schaible U. E. (2013). The Granuloma in Tuberculosis: Dynamics of a Host-Pathogen Collusion. Front. Immun. 3 (411), 1664–3224. 10.3389/fimmu.2012.00411 PMC3538277 23308075

[B15] Fenaroli F. Repnik U. Xu Y. Johann K. Van Herck S. Dey P. (2018). Enhanced Permeability and Retention-like Extravasation of Nanoparticles from the Vasculature into Tuberculosis Granulomas in Zebrafish and Mouse Models. ACS Nano 12 (8), 8646–8661. 10.1021/acsnano.8b04433 30081622

[B16] Feng H.-T. Zou S. Chen M. Xiong F. Lee M.-H. Fang L. (2020). Tuning Push-Pull Electronic Effects of AIEgens to Boost the Theranostic Efficacy for Colon Cancer. J. Am. Chem. Soc. 142 (26), 11442–11450. 10.1021/jacs.0c02434 32479068

[B17] Fernández-García M. Rey-Stolle F. Boccard J. Reddy V. P. García A. Cumming B. M. (2020). Comprehensive Examination of the Mouse Lung Metabolome Following *Mycobacterium tuberculosis* Infection Using a Multiplatform Mass Spectrometry Approach. J. Proteome Res. 19 (5), 2053–2070. 10.1021/acs.jproteome.9b00868 32285670 PMC7199213

[B18] Fu L. Wang Z. Dhankher O. P. Xing B. (2020). Nanotechnology as a New Sustainable Approach for Controlling Crop Diseases and Increasing Agricultural Production. J. Exp. Bot. 71 (2), 507–519. 10.1093/jxb/erz314 31270541

[B19] Gengenbacher M. Kaufmann S. H. E. (2012). *Mycobacterium tuberculosis*: success through Dormancy. FEMS Microbiol. Rev. 36 (3), 514–532. 10.1111/j.1574-6976.2012.00331.x 22320122 PMC3319523

[B20] Hakkimane S. Shenoy V. P. Gaonkar S. Bairy I. Guru B. R. (2018). Antimycobacterial Susceptibility Evaluation of Rifampicin and Isoniazid Benz-Hydrazone in Biodegradable Polymeric Nanoparticles against *Mycobacterium tuberculosis* H37Rv Strain. Ijn 13, 4303–4318. 10.2147/IJN.S163925 30087562 PMC6061404

[B21] Hein C. D. Liu X.-M. Wang D. (2008). Click Chemistry, a Powerful Tool for Pharmaceutical Sciences. Pharm. Res. 25 (10), 2216–2230. 10.1007/s11095-008-9616-1 18509602 PMC2562613

[B22] Herner A. Lin Q. (2016). Photo-triggered Click Chemistry for Biological Applications. Top. Curr. Chem. (Z) 374 (1), 1. 10.1021/acs.bioconjchem.7b0056210.1007/s41061-015-0002-2 PMC4935935 27397964

[B23] Kabir S. Junaid K. Rehman A. (2021). Variations in Rifampicin and Isoniazid Resistance Associated Genetic Mutations Among Drug Naïve and Recurrence Cases of Pulmonary Tuberculosis. Int. J. Infect. Dis. 103, 56–61. 10.1016/j.ijid.2020.11.007 33181327

[B24] Kiran D. Podell B. K. Chambers M. Basaraba R. J. (2016). Host-directed Therapy Targeting the *Mycobacterium tuberculosis* Granuloma: a Review. Semin. Immunopathol 38 (2), 167–183. 10.1007/s00281-015-0537-x 26510950 PMC4779125

[B25] Kumar A. Tan A. Wong J. Spagnoli J. C. Lam J. Blevins B. D. (2017). Nanotechnology for Neuroscience: Promising Approaches for Diagnostics, Therapeutics and Brain Activity Mapping. Adv. Funct. Mater. 27 (39), 1700489. 10.1002/adfm.201700489 30853878 PMC6404766

[B26] Le Droumaguet C. Wang C. Wang Q. (2010). Fluorogenic Click Reaction. Chem. Soc. Rev. 39 (4), 1233–1239. 10.1039/b901975h 20309483

[B27] Liao Y. Li B. Zhao Z. Fu Y. Tan Q. Li X. (2020). Targeted Theranostics for Tuberculosis: A Rifampicin-Loaded Aggregation-Induced Emission Carrier for Granulomas Tracking and Anti-infection. ACS Nano 14 (7), 8046–8058. 10.1021/acsnano.0c00586 32401009

[B28] Liu S. Su H. Bu L. Yan J. Li G. Huang J. (2021). Fluorogenic Probes for Mitochondria and Lysosomes via Intramolecular Photoclick Reaction. Analyst 146 (4), 1369–1375. 10.1039/d0an01982h 33393557

[B29] Magnussen A. Dinneen A. Ramesh P. (2013). Osteoarticular Tuberculosis: Increasing Incidence of a Difficult Clinical Diagnosis. Br. J. Gen. Pract. 63 (612), 385–386. 10.3399/bjgp13X669383 23834878 PMC3693798

[B30] Maiti B. Abramov A. Franco L. Puiggalí J. Enshaei H. Alemán C. (2020). Thermoresponsive Shape‐Memory Hydrogel Actuators Made by Phototriggered Click Chemistry. Adv. Funct. Mater. 30 (24), 2001683. 10.1002/adfm.202001683

[B31] Moosa M. S. Maartens G. Gunter H. Allie S. Chughlay M. F. Setshedi M. (2020). A Randomized Controlled Trial of Intravenous N-Acetylcysteine in the Management of Anti-tuberculosis Drug-Induced Liver Injury. Clin. Infect. Dis. 73, e3377–e3383. 10.1093/cid/ciaa1255 32845997

[B32] Muefong C. N. Sutherland J. S. (2020). Neutrophils in Tuberculosis-Associated Inflammation and Lung Pathology. Front. Immunol. 11, 962. 10.3389/fimmu.2020.00962 32536917 PMC7266980

[B33] Mukewar S. Mukewar S. Ravi R. Prasad A. Dua K. S. (2012). Colon Tuberculosis: Endoscopic Features and Prospective Endoscopic Follow-Up after Anti-tuberculosis Treatment. Clin. Transl. Gastroenterol. 3, e24. 10.1038/ctg.2012.19 23238066 PMC3491534

[B34] Nainar S. Kubota M. McNitt C. Tran C. Popik V. V. Spitale R. C. (2017). Temporal Labeling of Nascent RNA Using Photoclick Chemistry in Live Cells. J. Am. Chem. Soc. 139 (24), 8090–8093. 10.1021/jacs.7b03121 28562039

[B35] Nwe K. Brechbiel M. W. (2009). Growing Applications of “Click Chemistry” for Bioconjugation in Contemporary Biomedical Research. Cancer Biother. Radiopharm. 24 (3), 289–302. 10.1089/cbr.2008.0626 19538051 PMC2811415

[B36] Oehlers S. H. Cronan M. R. Scott N. R. Thomas M. I. Okuda K. S. Walton E. M. (2015). Interception of Host Angiogenic Signalling Limits Mycobacterial Growth. Nature 517 (7536), 612–615. 10.1038/nature13967 25470057 PMC4312197

[B37] Pai M. Behr M. A. Dowdy D. Dheda K. Divangahi M. Boehme C. C. (2016). Tuberculosis. Nat. Rev. Dis. Primers 2 (1), 16076. 10.1038/nrdp.2016.76 27784885

[B38] Pandey V. Chawla K. Acharya K. Rao S. Rao S. (2009). The Role of Polymerase Chain Reaction in the Management of Osteoarticular Tuberculosis. Int. Orthopaedics (Sicot) 33 (3), 801–805. 10.1007/s00264-007-0485-8 PMC2903087 18038134

[B39] Peng J. Samanta A. Zeng X. Han S. Wang L. Su D. (2017). Real-Time In Vivo Hepatotoxicity Monitoring through Chromophore-Conjugated Photon-Upconverting Nanoprobes. Angew. Chem. Int. Ed. Engl. 56 (15), 4165–4169. 10.1002/anie.201612020 28295935

[B40] Phillips R. O. Robert J. Abass K. M. Thompson W. Sarfo F. S. Wilson T. (2020). Rifampicin and Clarithromycin (Extended Release) versus Rifampicin and Streptomycin for Limited Buruli Ulcer Lesions: a Randomised, Open-Label, Non-inferiority Phase 3 Trial. The Lancet 395 (10232), 1259–1267. 10.1016/S0140-6736(20)30047-7 PMC7181188 32171422

[B41] Pigrau-Serrallach C. Rodríguez-Pardo D. (2013). Bone and Joint Tuberculosis. Eur. Spine J. 22 (4), 556–566. 10.1007/s00586-012-2331-y PMC3691411 22711012

[B42] Ramakrishnan L. (2012). Revisiting the Role of the Granuloma in Tuberculosis. Nat. Rev. Immunol. 12 (5), 352–366. 10.1038/nri3211 22517424

[B43] Rodriguez-Takeuchi S. Y. Renjifo M. E. Medina F. J. (2019). Extrapulmonary Tuberculosis: Pathophysiology and Imaging Findings. RadioGraphics 39 (7), 2023–2037. 10.1148/rg.2019190109 31697616

[B44] Rothchild A. C. Olson G. S. Nemeth J. Amon L. M. Mai D. Gold E. S. (2019). Alveolar Macrophages Generate a Noncanonical NRF2-Driven Transcriptional Response to *Mycobacterium tuberculosis In Vivo* . Sci. Immunol. 4 (37), e6693. 10.1126/sciimmunol.aaw6693 PMC6910245 31350281

[B45] Sarathy J. P. Zuccotto F. Hsinpin H. Sandberg L. Via L. E. Marriner G. A. (2016). Prediction of Drug Penetration in Tuberculosis Lesions. ACS Infect. Dis. 2 (8), 552–563. 10.1021/acsinfecdis.6b00051 27626295 PMC5028112

[B46] Seres T. Knickelbein R. G. Warshaw J. B. Johnston R. B. Jr. (2000). The Phagocytosis-Associated Respiratory Burst in Human Monocytes Is Associated with Increased Uptake of Glutathione. J. Immunol. 165 (6), 3333–3340. 10.4049/jimmunol.165.6.3333 10975851

[B47] Shang X. Lai R. Song X. Li H. Niu W. Guo J. (2017). Improved Photoinduced Fluorogenic Alkene-Tetrazole Reaction for Protein Labeling. Bioconjug. Chem. 28 (11), 2859–2864. 10.1021/acs.bioconjchem.7b00562 29022697 PMC5688003

[B48] Shrivastava N. Singh P. Nayak B. Garg B. (2019). The Spectrum of Clinical and Urodynamic Findings in Patients with Spinal Tuberculosis Exhibiting Lower Urinary Tract Symptoms, before and after Spinal Surgical Intervention with Antitubercular Treatment: A Prospective Study. Asian Spine J. 13 (4), 615–620. 10.31616/asj.2018.0217 30909676 PMC6680043

[B49] Singh M. Vaughn C. Sasaninia K. Yeh C. Mehta D. Khieran I. (2020). Understanding the Relationship between Glutathione, TGF-β, and Vitamin D in Combating *Mycobacterium tuberculosis* Infections. Jcm 9 (9), 2757. 10.3390/jcm9092757 PMC7563738 32858837

[B50] Sterling T. R. Njie G. Zenner D. Cohn D. L. Reves R. Ahmed A. (20202020). Guidelines for the Treatment of Latent Tuberculosis Infection: Recommendations from the National Tuberculosis Controllers Association and CDC, 2020. Am. J. Transpl. 20 (4), 1196–1206. 10.1111/ajt.15841 PMC7041302 32053584

[B51] Venketaraman V. Dayaram Y. K. Amin A. G. Ngo R. Green R. M. Talaue M. T. (2003). Role of Glutathione in Macrophage Control of Mycobacteria. Infect. Immun. 71 (4), 1864–1871. 10.1128/IAI.71.4.1864-1871.2003 12654802 PMC152031

[B52] Venketaraman V. Dayaram Y. K. Talaue M. T. Connell N. D. (2005). Glutathione and Nitrosoglutathione in Macrophage Defense against mycobacterium Tuberculosis. Infect. Immun. 73 (3), 1886–1889. 10.1128/IAI.73.3.1886-1889.2005 15731094 PMC1064956

[B53] Villa S. Ferrarese M. Sotgiu G. Castellotti P. F. Saderi L. Grecchi C. (2020). Latent Tuberculosis Infection Treatment Completion while Shifting Prescription from Isoniazid-Only to Rifampicin-Containing Regimens: A Two-Decade Experience in Milan, Italy. Jcm 9 (1), 101–113. 10.3390/jcm9010101 PMC7019895 31906078

[B54] Wang Q. Guan J. Wan J. Li Z. (2020). Disulfide Based Prodrugs for Cancer Therapy. RSC Adv. 10 (41), 24397–24409. 10.1039/D0RA04155F PMC9055211 35516223

[B55] Wu Y. Guo G. Zheng J. Xing D. Zhang T. (2019). Fluorogenic "Photoclick" Labeling and Imaging of DNA with Coumarin-Fused Tetrazole *In Vivo* . ACS Sens. 4 (1), 44–51. 10.1021/acssensors.8b00565 30540170

[B56] Xu W. Snell L. M. Guo M. Boukhaled G. Macleod B. L. Li M. (2021). Early Innate and Adaptive Immune Perturbations Determine Long-Term Severity of Chronic Virus and *Mycobacterium tuberculosis* Coinfection. Immunity 54 (3), 526–541. 10.1016/j.immuni.2021.01.003 33515487 PMC7946746

[B57] Yang L. Qu W. Zhang X. Hang Y. Hua J. (2015). Constructing a FRET-Based Molecular Chemodosimeter for Cysteine over Homocysteine and Glutathione by Naphthalimide and Phenazine Derivatives. Analyst 140 (1), 182–189. 10.1039/C4AN01732C 25407553

[B58] Zhang R. Zheng J. Zhang T. (2020). *In Vivo* selective Imaging of Metabolic Glycosylation with a Tetrazine-Modified Upconversion Nanoprobe. RSC Adv. 10, 15990–15996. 10.1039/D0RA01832E PMC9052955 35493688

[B59] Zheng J. Wu Y. Xing D. Zhang T. (2019a). Synchronous Detection of Glutathione/hydrogen Peroxide for Monitoring Redox Status *In Vivo* with a Ratiometric Upconverting Nanoprobe. Nano Res. 12 (4), 931–938. 10.1007/s12274-019-2327-6

[B60] Zheng J. Zeng Q. Zhang R. Xing D. Zhang T. (2019b). Dynamic-Reversible Photoacoustic Probe for Continuous Ratiometric Sensing and Imaging of Redox Status *In Vivo* . J. Am. Chem. Soc. 141 (49), 19226–19230. 10.1021/jacs.9b10353 31770490

[B61] Zheng J. Zhan Q. Jiang L. Xing D. Zhang T. Wong K.-L. (2020). A Bioorthogonal Time-Resolved Luminogenic Probe for Metabolic Labelling and Imaging of Glycans. Inorg. Chem. Front. 7, 4062–4069. 10.1039/D0QI00728E

[B62] Zhou M. Hu J. Zheng M. Song Q. Li J. Zhang Y. (2016). Photo-click Construction of a Targetable and Activatable Two-Photon Probe Imaging Protease in Apoptosis. Chem. Commun. 52 (11), 2342–2345. 10.1039/C5CC09973K 26729240

[B63] Zwerling A. (2020). Understanding Spending Trends for Tuberculosis. Lancet Infect. Dis. 20 (8), 879–880. 10.1016/S1473-3099(20)30316-9 32334657 PMC7180033

