# Supplementary material for: Photoclick Reaction Constructs Glutathione-Responsive Theranostic System for Anti-Tuberculosis
Source: Front Mol Biosci. 2022 Feb 14;9:845179. doi: 10.3389/fmolb.2022.845179 (PMC8883117; doi:10.3389/fmolb.2022.845179)
Supplement: Supplementary file 1 [file DataSheet1.docx]

Figures and Caption

Photoclick Reaction Constructs Glutathione-Responsive Theranostic System for Anti-Tuberculosis

Jundun Zheng^2,‡^, Xun Long^1,‡^, Hao Chen^3,‡^, Zhisheng Ji^4,‡^, Bowen Shu^2^, Rui Yue^2^, Yechun Liao^1^, Shengchao Ma^7*^, Kun Qiao^6*^, Ying Liu^1^* and Yuhui Liao^2,5,7*^

^1^Department of Science and Education, The Third People’s Hospital of Bijie City, Bijie, 551700 (P.R. China).

^2^Molecular Diagnosis and Treatment Center for Infectious Diseases, Dermatology Hospital, Southern Medical University, Guangzhou 510091 (P.R. China).

^3^Division of Gastrointestinal Surgery, Department of General Surgery, Nanfang Hospital, Southern Medical University, Guangzhou 510091 (P.R. China).

^4^Department of Orthopedics, the First Affiliated Hospital of Jinan University, Jinan University, Guangzhou 510630 (P.R. China).

^5^Department of Infectious Disease, the Fifth Affiliated Hospital, Sun Yat-sen University, Guangzhou 510091 (P.R. China).

^6^Department of Thoracic Surgery, Shenzhen Third People’s Hospital, Shenzhen 518110, (P.R. China).

^7^NHC Key Laboratory of Metabolic Cardiovascular Diseases Research, Ningxia Key Laboratory of Vascular Injury and Repair Research, Ningxia Medical University, Yinchuan 750004, China

‡ These authors contributed equally.

*** Correspondence:**

Corresponding Author

Shengchao Ma, E-mail: [solarmsc@163.com](mailto:solarmsc@163.com)

Kun Qiao, E-mail: [szqiaokun@163.com](mailto:szqiaokun@163.com)

Ying Liu, E-mail: [LX13985359719@163.com](mailto:LX13985359719@163.com)

Yuhui Liao, E-mail: [liaoyh8@mail.sysu.edu.cn](mailto:liaoyh8@mail.sysu.edu.cn)

Keywords: Tuberculosis_1_, Theranostic System_2_, Photoclick Reaction_3_, Microenvironment_4_, Glutathione_5_.

Figure S1. Synthetic route of HA-Cys-MA.





Figure S2. Synthetic route of HA-Lys-Tet.


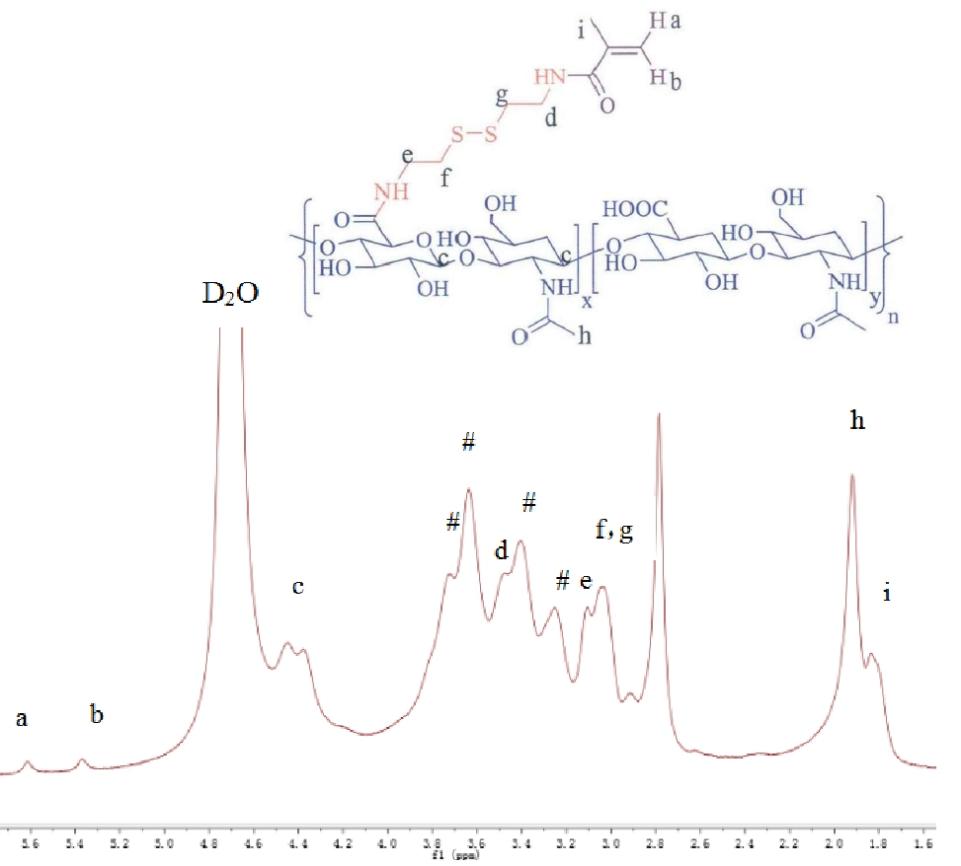


Figure S3. The 1H-NMR of HA-Cys-MA.


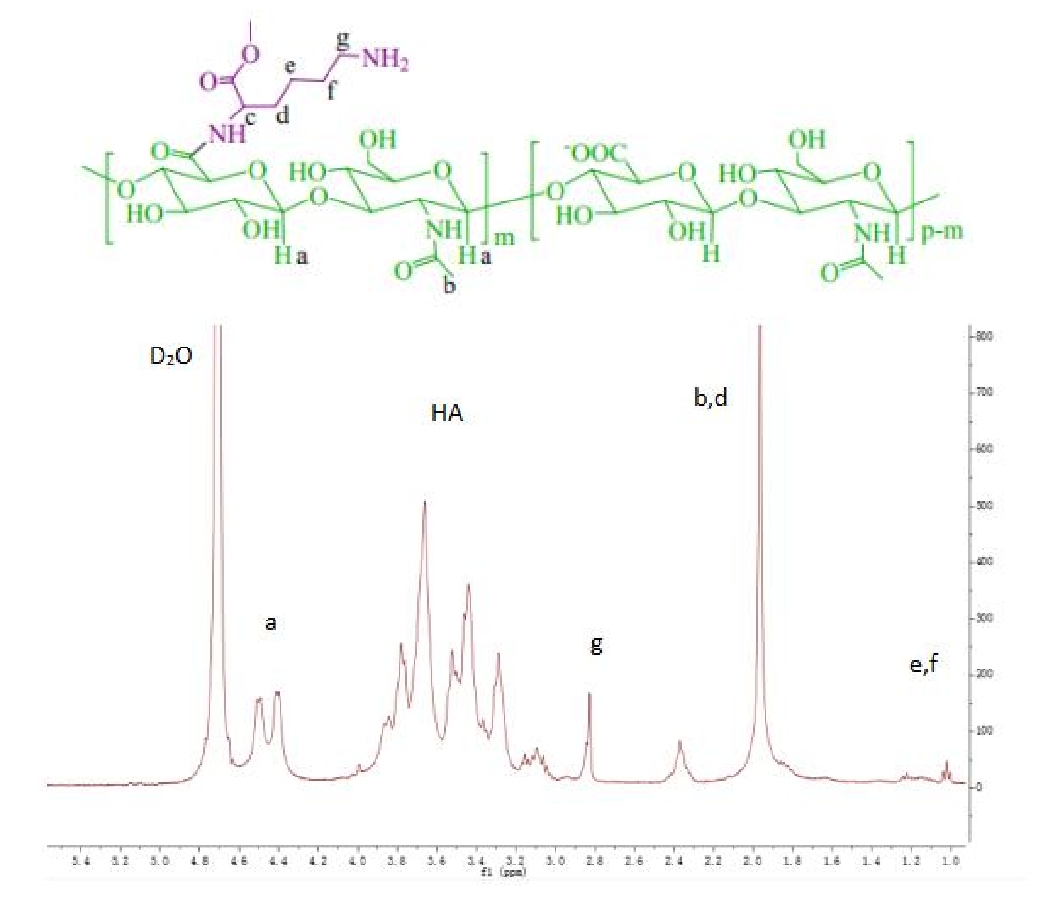


Figure S4. The 1H-NMR of HA-Lys-NH_2_.


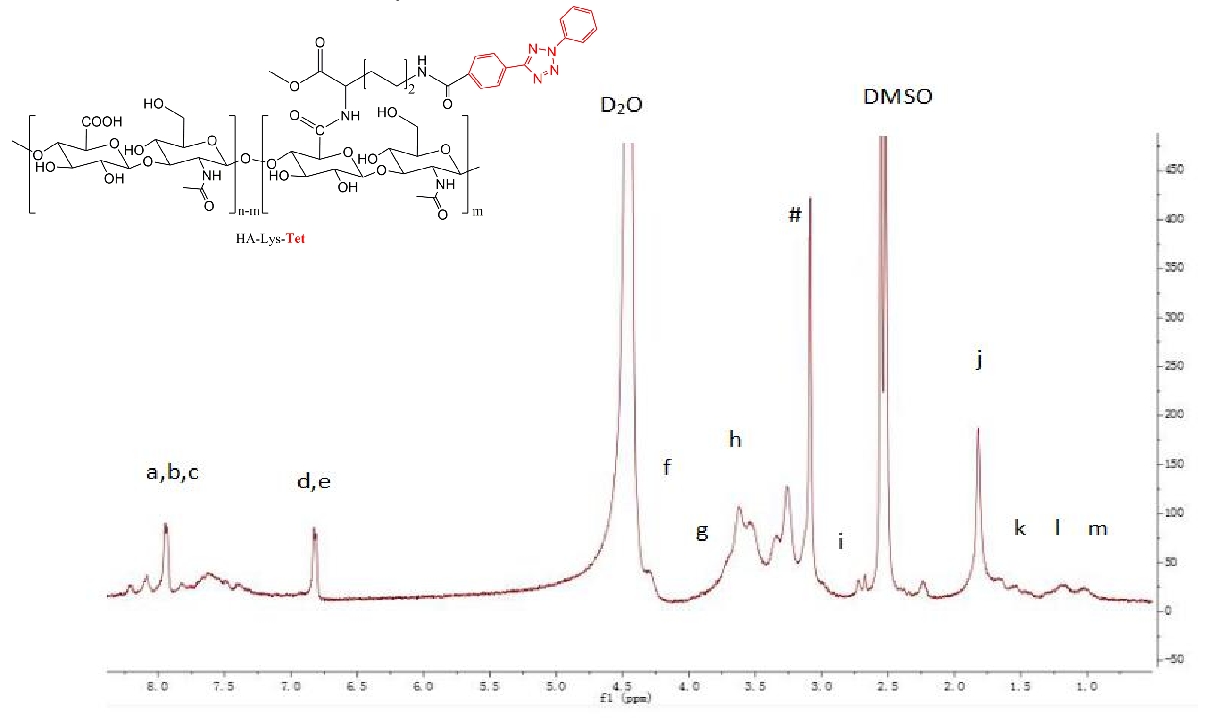


Figure S5. The 1H-NMR of HA-Lys-Tet.

**Reference**

[1] Y. Liao, B. Li, Z. Zhao, Y. Fu, Q. Tan, X. Li, W. Wang, J. Yin, H. Shan, B. Z. Tang, X. Huang, *ACS Nano* **2020**, *14*, 8046-8058
